# Long‐Term Care at Advanced Ages: The Effect of Spousal Bereavement on Institutional Care Needs

**DOI:** 10.1002/hec.70043

**Published:** 2025-10-13

**Authors:** Chantal Schouwenaar, Pierre Koning, Yvonne Krabbe‐Alkemade, Maarten Lindeboom, France Portrait

**Affiliations:** ^1^ School of Business and Economics Vrije Universiteit Amsterdam Amsterdam the Netherlands; ^2^ Research Fellow Tinbergen Institute Amsterdam the Netherlands; ^3^ Research Fellow Institute of Labor Economics Bonn Germany; ^4^ Center for Health Economics Monash University Melbourne Australia

**Keywords:** aging, causality, dynamic treatment effect models, elderly, entitlements, health data, old age, panel data, senior citizens, spouse

## Abstract

With populations aging, long‐term care (LTC) needs and costs rise, intensifying the reliance on informal care. Since spouses predominantly provide such care, it is crucial to understand the transition dynamics from widowhood to institutional LTC, which is the main driver of the public LTC costs in most OECD countries. Our study examines the causal effect of widowhood on the onset of needs for institutional LTC. For this we use recently developed event‐time models that accommodate for variation in treatment time and dynamic effects of treatment. Our results show that partner loss significantly increases the need for institutional LTC. The average effect of partner loss on the need for institutional LTC peaks at a 1.5 percentage point rise 3 months after widowhood and disappears after 10 months. The effect is strongest for individuals with psycho‐geriatric disorders, the oldest old and the frail. Addressing the immediate need for institutional LTC after widowhood remains critical for effective aging‐in‐place strategies.

**Abbreviations**
ATT: Average Treatment effect on the Treated
DiD: difference‐in‐differences
HIa: Health Insurance act
LTC: long‐term care
LTCa: Long‐Term Care act
SSa: Social Support act

## 1 Introduction

As the global population ages, there is a continuing rise in the likelihood of developing chronic diseases and frailty among older adults (OECD 2023a). This will probably require an increase in the provision of long‐term care (LTC) services (OECD 2023c). The initial care burden often falls on the shoulders of family members, supported by formal home care as necessary (Rocard and Llena‐Nozal 2022).^1^ When informal care possibly combined with formal home care is insufficient to meet the need for LTC, more costly institutional LTC may be needed. This institutional LTC is the main driver of public LTC costs in most OECD countries (OECD 2023c).^2^ With increasing LTC costs and a growing shortage of formal caregivers, the trade‐off between combining informal and formal home care on the one hand and the more expensive institutional LTC on the other hand, is therefore one of the key policy issues in most developed countries (OECD 2023b). In this paper, we address this trade‐off by examining the causal effect of the death of a partner, the most important informal care provider, on the onset of needs for institutional LTC.

### 1.1 The Role of Informal Care

Informal care refers to support provided by family, friends, and social networks to individuals needing help with daily activities (OECD 2023b). A recent study across 19 OECD countries, shows that around 60% of older adults depend on informal care, with 13% of people aged 50 years and older providing such care weekly and 8% daily (Rocard and Llena‐Nozal 2022). Informal care includes not only household tasks or transportation, but also more intensive activities such as personal care and emotional support (Broese van Groenou and de Boer 2016). Throughout the years, studies have consistently shown that spouses and adult children—particularly women aged 50–75—remain the primary caregivers and that partners frequently provide the most intensive and irreplaceable forms of care (Pinquart and Sörensen 2011; Rocard and Llena‐Nozal 2022). Children and non‐family caregivers often face barriers—like time constraints, geographic distance, and lack of expertise—that may limit their caregiving capacity compared to partners (Korfhage and Fischer‐Weckemann 2024; Pinquart and Sörensen 2011). This is confirmed by our own analysis of data from the Longitudinal Aging Study Amsterdam, which shows that partners provide about 80% of personal and nursing informal care, while children help more often with household tasks and administrative matters (see Table A1 of Appendix A).

Recent LTC strategies increasingly lean on informal support networks while promoting the “aging‐in‐place” approach (Bom et al. 2022; Bakx et al. 2023; van der Burg et al. 2020; European Commission 2018). Aging‐in‐place entails equipping older adults with sufficient assistance to continue living autonomously in their own homes for as long as feasible (van Boekel et al. 2021; Pani‐Harreman et al. 2021). Evidence shows that the majority of older individuals favor this arrangement over nursing home residence because it preserves autonomy, independence and personal dignity (Stones and Gullifer 2016). Successful “aging‐in‐place” typically relies on informal care provision, especially on the informal care provided by the partner (Rocard and Llena‐Nozal 2022). This implies that the partner's death could lead to an increase in institutional LTC needs.

### 1.2 Partner Loss and Institutional LTC Needs

**FIGURE 1:**
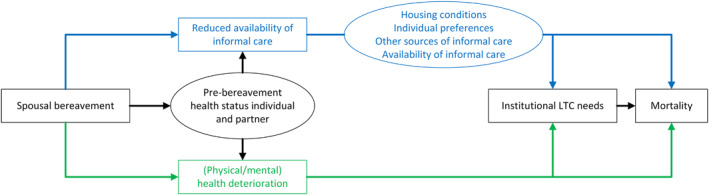
Potential mechanisms following spousal bereavement on round‐the‐clock care needs.

The effect of partner loss on the onset of needs for institutional LTC can be twofold. This is illustrated with the Directed Acyclic Graph (DAG) diagram shown in Figure 1. First, spousal bereavement can directly and mechanically reduce the availability of informal care, particularly when the deceased partner was in relatively good health and was able to provide such care prior to death. This is especially relevant for psycho‐geriatric care (PG) (such as dementia), which often requires 24/7 assistance that formal home care cannot provide (Korhonen et al. 2018). The extent to which partner loss directly leads to institutional LTC needs is moderated by factors such as the availability of alternative informal care sources and individual preferences (Amankour et al. 2025). This mechanism is illustrated in the upper, blue section of Figure 1. Second, spousal bereavement—especially in the context of pre‐existing frailty—may lead to significant mental and physical health deterioration of the surviving partner, thereby further increasing the likelihood of institutional care needs. This constitutes a more indirect pathway from spousal bereavement to additional LTC needs, as depicted in the lower part of the DAG depicted in green. Indeed, studies show that widowhood is related not only to poorer emotional health, but also to worse cognitive and physical health, and increased health care costs, for example, (Bakx et al. 2013; Moon et al. 2014; Pena‐Longobardo et al. 2021; Rolden et al. 2014; Carey et al. 2014; Ghesquiere et al. 2016; van Boekel et al. 2021; Vedder et al. 2022; Zhao et al. 2021; Ornstein et al. 2019). Although partner loss could increase the need for institutional LTC, it is also plausible that heightened mortality rates among the widowed compared to non‐widowed individuals (van den Berg et al. 2011) may reduce their overall time spent in nursing homes. This creates an intriguing interplay between increased needs for institutional LTC due to partner loss and the offsetting effect of mortality.

Numerous empirical studies have examined how informal care influences transitions into institutional care. For instance, Syse et al. (2022) found that older Norwegians without partners or children were more likely to enter institutional LTC, while those with supportive family had the lowest likelihood. Hanaoka and Norton (2008) noted that the effect of children varies depending on gender, marital status, and the children's opportunity costs. To our knowledge, the number of studies examining the role of children and other sources of informal care after spousal bereavement is limited. An exception is the study by Noël‐Miller (2010) which showed, using a sample of older American couples, that adult children helped their mother stay out of a nursing home, regardless of whether their father was still alive. In the case of fathers, however, children delayed institutionalization only after the mother had passed away.

### 1.3 Robust Causal Analysis

To assess the causal effect of widowhood on the transition to institutional LTC, this paper uses recently developed event‐time methods that account for variation in treatment time (i.e., the timing of partner's death) and allow assessment of dynamic effects of the treatment (Callaway and Sant’Anna 2021b). These methods also control for the potentially selective nature of the treatment and the biasing impact this may have on the causal effects. We apply these methods on population‐wide, individual‐level, Dutch administrative data of older individuals (75+) that include information on applications for access to institutional LTC from January 2018 to December 2019. At this point, it is important to stress that entitlements for institutional care in the Netherlands do not depend on the (un)availability of partners who are able to provide informal care. The treatment effects due to reduced informal care in our analysis therefore represent the effects of latent demand for formal care that is no longer provided by the partner. We enrich these data with individual‐level administrative information on partner status, home care usage, and mortality. The data also allow to differentiate between different types of institutional LTC, namely care related to physical (somatic) impairments and to cognitive and/or PG impairments. PG institutional LTC is more costly, since those patients stay on average longer in nursing homes (ten Koppel et al. 2023).^3^

We find immediate effects of widowhood on the need for institutional LTC. The effects on institutional LTC needs are limited to the first 10 months following partner loss. They are most pronounced for PG needs during the first 4 months after widowhood than for somatic needs. However, the effect after widowhood last longer for the ones with somatic needs. Furthermore, the effect is most substantial for individuals who received home care prior to their partner's death and also for the oldest old.^4^ This suggests that widowhood predominantly affects partners who would have probably received an entitlement for institutional LTC later on. Perhaps surprisingly, the magnitude of the effect does not differ by sex.

### 1.4 Contribution to Literature

Our study relates to and expands upon work that investigates the determinants of LTC, in particular institutional LTC. First, earlier research focused on the actual use of (institutional) LTC as a proxy for needs for care. For instance, van Houtven and Norton (2004) and Bakx et al. (2013) examine the effect of the presence of potential informal care providers on actual use of formal and informal LTC. van der Burg et al. (2020) look at LTC use after a stroke or femoral fracture and find that having a spouse is associated with a decrease formal home care and institutional care use. Closer to our analysis, van Boekel et al. 2021 Rolden et al. (2014); Ornstein et al. (2019) and Pena‐Longobardo et al. (2021) examine the effect of spousal loss on health, well‐being, or care use. However, actual use of care is affected by factors such as individual preferences and local availability of care. In this paper, we focus on eligibility for institutional LTC instead of care use which allows to more accurately study the development of needs for institutional LTC after widowhood.

Second, from a methodological perspective, we are the first to examine dynamic causal effects of partner loss on the onset of needs for institutional LTC. Some of the referenced papers which also aim at assessing causal effects use instrumental variable methods, for example, van Houtven and Norton (2004), and Bergeot and Tenand (2023) or classical differences‐in‐differences (DiD) methods that compare groups who lose their partner with those who remain married. These “controls” are likely to be healthier and/or differ in other characteristics from those who have experienced partner loss. The protective effect of having a partner has been demonstrated recently by Kulu et al. (2024). As the recent literature (Callaway and Sant’Anna 2021b) has shown, effects estimates of these models can be seriously biased if the bereavement effects vary over time. We apply the method by Callaway and Sant’Anna (2021b) on a sample of individuals who all lose their partner during the observation period. In this way, the treated (those who lose their partner at a specific point in time) are compared with those who are treated in the future (the controls). The controls are much more similar to the treated and the method only requires the less restrictive assumption of randomness in the actual timing of the treatment.

## 2 Contextual Background

This study focuses on the Netherlands. Although the share of older Dutch individuals is comparable to most developed countries, the Netherlands spends the highest percentage of GDP on LTC among OECD countries. In 2021, 4.4% of the Dutch GDP was spent on LTC, with 80% of that expenditure specifically directed toward institutional LTC (OECD 2023c).

Since 2015, the Dutch government has implemented substantial reforms to the LTC system, aiming primarily to better control expenditures. The reform of 2015 led to a substantial increase in the decentralization of authority and the establishment of a more multi‐tiered governance structure. In 2015, there was a moderate decrease in expenditures on LTC for individuals aged 65 and older, but this lasted only for a period of 1 year (Krabbe‐Alkemade et al. 2020). Since then, LTC expenditures have continued to increase substantially (Bakx et al. 2023).

The reform also promoted aging‐in‐place and informal care has become indispensable in the provision of LTC in the Netherlands. In 2019, approximately 19% of Dutch individuals aged 75 and older received informal care, of whom one third also received formal care (de Boer et al. 2020). The care pathway of a frail older individual, who is increasingly dependent on informal and formal LTC due to somatic and/or PG disabilities, can be characterized by three main stages (Ministry of Public Health and Welfare and Sport 2016).^5^

In the first stage, when an older person is no longer able to independently manage the household due to health limitations, social assistance can be requested from the municipality. The municipality may then provide general facilities (community centers, grocery services) or individually tailored services, such as household chores or transportation. This form of social LTC is organized in compliance with the Social Support act (SSa). The municipality first examines the contributions of potential informal caregivers to the individual's care and then determines the level of formal support required (Ministry of Public Health and Welfare and Sport 2016).

In the second stage, an older person requires more care than just social LTC, and needs more specialized, medical personal care. The provision of such care is administered by the Health Insurance act (HIa). Similarly to SSa care, an entitlement is required to have access to this type of care. A district nurse assesses the care needs, placing emphasis on self‐sufficiency and personal autonomy. In addition, the district nurse assesses the capacity of the older person's social network and determines the care provided accordingly (Ministry of Public Health and Welfare and Sport 2016).

In the final stage, the individual needs extensive and permanent care. When living independently is no longer feasible, the individual may ask for publicly‐insured round‐the‐clock LTC.^6^ The decision is often not made deliberately by the older adult; instead, the request is frequently initiated by a healthcare professional, such as a general practitioner or community nurse. Round‐the‐clock care is most often provided in a nursing home, but can also, under specific circumstances, be provided at home.^7^ The provision of this type of LTC is arranged in compliance with the Long‐term Care act (LTCa) (Ministry of Public Health and Welfare and Sport 2016). This is the care that we are investigating in this study (from now on referred to as “institutional LTC”).

Access to institutional LTC is granted by a governmental independent organization, known as the Care Needs Assessment Center (CNAC). The CNAC examines whether applicants are eligible for this type of care, using a set of nationwide, objective health‐related criteria (this does not include the availability of a partner or other family members). The evaluation by the CNAC to determine whether applicants are eligible can take up to 6 weeks. Requests can be rejected and only individuals with a valid CNAC entitlement have access to LTCa care. Regional care offices are responsible for facilitating the actual use of LTCa care. The vast majority of the LTCa entitlements is given to older people with a severe somatic and/or a PG disability (Ministry of Public Health and Welfare and Sport 2016; ten Koppel et al. 2023).^8^ The different types and tariffs of entitlements for institutional LTC are described in Table 1. The Dutch tariffs are the highest among OECD countries. Specifically, tariffs for institutional care for older individuals with severe needs exceed four times the Dutch median income—compared to an OECD average of twice the national median (OECD 2023a). These high rates are partly due to high salaries.

**TABLE 1:** Entitlements and daily rates for round‐the‐clock LTC in the Netherlands in 2019.

Care profiles	Description	Care in nursing homes		Care at home	
		€/day	# Of ind.	€/day	# Of ind.
VV4	Sheltered housing with intensive guidance and extensive care	197.30	22,585	156.40	2765
VV5	Protected living with intensive dementia care	250.10	58,640	208.50	4355
VV6	Protected living with intensive care and nursing	251.10	26,555	208.80	1685
VV7	Protected living with very intensive care due to specific conditions with an emphasis on guidance	293.20	10,845	248.60	165
VV8	Protected living with very intensive care due to specific conditions with an emphasis on care/nursing	331.30	2090	282.50	55

^*a*^: *Source:* Nederlandse Zorgautoriteit (2019).

## 3 Methods

### 3.1 Data Sources

We combine longitudinal, individual‐level data from various administrative sources from Statistics Netherlands (CBS 2023). These data provide daily information from January 2018 up to December 2019 and include information on: (1) applications and entitlements for institutional LTC; (2) survival status; (3) demographic characteristics; (4) presence of children; and, (5) use of formal home care provided by a district nurse, and financed through the HIa. From here on, we will refer to this as home care.

### 3.2 Study Population

The initial dataset consists of all individuals living in the Netherlands on January 1, 2018 (N ≈ 17 million). From this we select all individuals between ages 75 and 100 (N ≈ 1,6 million).^9^ We next select all individuals registered as living with someone. This is most likely a spouse or a registered partner (*N* = 666,267).^10^ From here on, we will refer to the spouse or registered partner as the partner. We subsequently select those who lost their partner at some point between January 1, 2018 and December 31, 2019 (*N* = 78,606).^11^ Finally, we exclude individuals who already were entitled to institutional LTC on January 1, 2018. The final study sample consists of 48,996 individuals.

In the analyses we construct monthly observations for each individual and follow them from January 2018 until December 2019, or until the time of their death if this occurs before December 2019. 6.6% of the individuals in our sample die before the end of the observation period. We thus have an unbalanced panel dataset, with a maximum of 24 successive monthly observations per individual. We define the treatment as the death of the partner.

### 3.3 Dependent Variables

Being granted institutional LTC is our main outcome variable, which we describe using three binary variables: (1) Being granted institutional LTC; (2) Being granted PG institutional LTC; and (3) Being granted somatic institutional LTC.

Table 2 illustrates how the main outcome variable is constructed for two fictitious individuals. The individual with Id =1 is still alive at the end of the observational period. Id =1 was granted institutional LTC in April 2018. Id =2 was granted institutional LTC in February 2018 and died in May 2018.

**TABLE 2:** Dependent variable construction for two fictitious individuals.

Id	Calendar time	Timing of entitlement	Timing of death	Entitlement variable
1	January 2018	April 2018	*missing*	0
1	February 2018	April 2018	*missing*	0
1	March 2018	April 2018	*missing*	0
1	April 2018	April 2018	*missing*	1
1	May 2018	April 2018	*missing*	1
1	June 2018	April 2018	*missing*	1
—	—	—	—	—
2	January 2018	February 2018	May 2018	0
2	February 2018	February 2018	May 2018	1
2	March 2018	February 2018	May 2018	1
2	April 2018	February 2018	May 2018	1
2	May 2018	February 2018	May 2018	*missing*
2	June 2018	February 2018	May 2018	*missing*
—	—	—	—	—

### 3.4 Independent Variables

The main independent variable is a binary variable for the month of death of the partner. We control for—and will stratify on—several variables. Beside age and gender, we include the number of children alive at the beginning of the observation period to control for another potential source of informal care. Home care use is also included as a dummy variable indicating whether the individual received home care somewhere in January 2018^12^ and can be viewed as a proxy for the individual's initial health condition (Jorm et al. 2010). Finally, we use the average percentage of high incomes on the 4‐digit postal code of residence to proxy the socioeconomic status of the individual (below 25%; 25%–50%; 50% or more; or missing).^13^

Table 3 presents the demographic, socioeconomic and health characteristics of all individuals in our sample at baseline, 11.7% of whom received an entitlement (i.e., a successful application) for institutional LTC during the observation window. For males, this is 12.4%, while for females this is 11.4%. About 64% of our sample is female. The fraction of individuals with an institutional LTC entitlement increases with age. Table 3 also indicates a higher prevalence of institutional LTC entitlements for those who have already used other sources of care. Those having one, two or three children alive at the beginning of 2018 have on average lower LTC entitlement rates than those without children, while this is slightly higher for those with four or more children. On average, LTC entitlement fractions are lower for individuals living in a neighborhood with a higher average income.

**TABLE 3:** Sample characteristics.

	Institutional LTC entitlement	No institutional LTC entitlement	Total
	%	%	
Sex			
Male	12.4	87.6	17,629
Female	11.4	88.6	31,368
Age in years at 1/1/2018			
75–79	5.5	94.5	19,926
80–84	11.2	88.8	17,135
85–89	20.4	79.6	9443
90–94	31.4	68.6	2300
95–100	43.5	56.5	193
Homecare use in 2018–2019			
No	2.4	97.6	26,063
Yes	22.4	77.6	22,934
Children alive at 1/1/2018			
0	13.2	86.8	3840
1	12.2	87.8	6816
2	10.5	89.5	18,858
3	11.4	88.6	11,574
4 or more	14.0	86.0	7909
Socio‐economic status at 1/1/2018			
Missing	11.6	88.4	4913
0%–25%	11.8	88.2	30,674
25%–50%	11.8	88.2	13,242
50%–100%	8.3	91.7	168
Total	11.7	88.3	48,997

**FIGURE 2:**
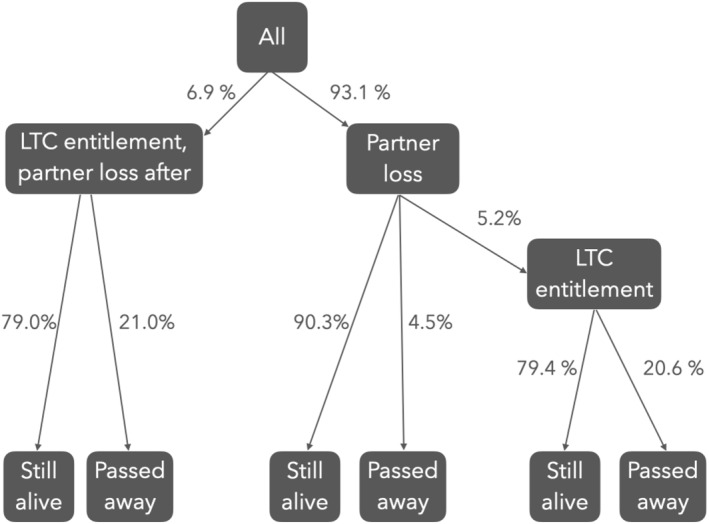
Possible transitions of the individuals in our sample.

Figure 2 shows the evolution of the partner, entitlement and survival status of all individuals included in our sample during the observation period. 6.9% of the individuals in our study sample receive an entitlement for institutional LTC before partner loss and 5.2% after. Moreover, 6.6% of all individuals died before December 2019.

### 3.5 Attrition Due to Mortality

**FIGURE 3:**
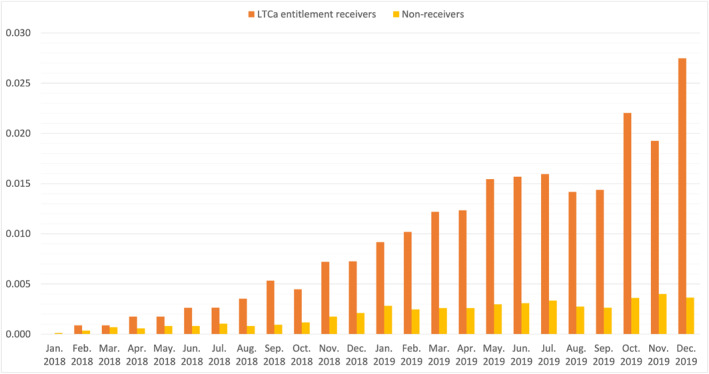
Monthly individuals' mortality rates in selected sample.

Since mortality in our sample only occurs after partner loss and roughly the same number of individuals lose their partner each month, mortality rates are low in the beginning of the observational window and relatively high at the end. This is mirrored in Figure 3, which shows the distribution of monthly mortality rates stratified by those who have and have not been granted institutional LTC entitlement.

As we will argue later on in more detail, our estimation method compares “treated” individuals (i.e., those who lose their partner) with “not‐yet‐treated” individuals (i.e., those who will experience partner loss later during our observation period).^14^ As time proceeds there is mortality selection in the data that will change the composition of the survivors in the sample and consequently make “not‐yet treated” not comparable to the “currently” treated, and hence will bias the treatment effect. We therefore control effect estimates of partner loss for biases stemming from selective mortality.

### 3.6 Empirical Model

We estimate an event‐time linear probability model with lead and lag effects on the likelihood of institutional LTC entitlement, measured before and after the month of partner loss. This setup allows for differential timing of partner loss during the observation period and treatment dynamics of the effect of partner loss in subsequent months. The model specification follows the so‐called staggered Difference‐in‐Difference set‐up of Callaway and Sant’Anna (2021b):

(1) $$Y_{i,s,t}=X_{i}\beta +\sum_{k=-23}^{-2}\;\;\gamma_{k}^{lead}\times I_{i,k}+\sum_{k=0}^{23}\;\;\gamma_{k}^{lags}\times I_{i,k}+\phi_{s}+\epsilon_{is},$$

$Y_{i,s,t}$ represents a variable that indicates whether individual i who experienced partner loss in calendar month t has entitlement for institutional LTC at calendar time s (s = 1,..24).^15^ Institutional LTC entitlement may have been received in months before time s (or t) and LTC entitlement remains until the individual dies. Stated differently, the outcome variable Y is an absorbing state that remains equal to one in all months following the award of institutional LTC (see Table 2 for the construction of $Y_{i,s,t}$). β denotes the impact of covariates X to allow for individual heterogeneity.^16^ X includes age, gender, SES, the number of children still alive and whether the individual receives home care.^17^ $I_{i,k}$ is an indicator for individual i that is equal to one when being k periods away from the initial treatment at time t, namely the partner's death, and zero otherwise. The maximum time period before the treatment is 23 months for those who lose their partner in the last month (December 2019), and the maximum time period after partner loss equals 23 months for those who lose their partner in the first month (January 2018). The effect sizes are normalized to zero at k=−1 (the month prior to partner loss). Finally, ϕ represents the effect of calendar time effects and $\epsilon_{is}$ is an error term that is assumed i.i.d. with a mean of zero and variance of $\sigma^{2}$.

The second term on the right‐hand side of Equation (1) allows for potential anticipation effects *before* partner loss. Specifically, the parameters $\gamma_{k}^{lead}$ measure the effect on $Y_{i,s,t}$ in months before the actual treatment takes place. Absence of any anticipation effects would imply $\gamma_{k}^{lead}=0$ for all k<−1 (and s<t). Similarly, the third term on the right‐hand side of the equation captures treatment dynamics $\left(\gamma_{k}^{lags}\right)$ with indicators $I_{i,k}$ being equal to one when the actual month s is k months *after* the month of partner loss, and zero otherwise. The corresponding values of $\gamma_{k}^{lags}$ display the effect on the likelihood of having received entitlement for LTC k months since partner loss. Since LTC entitlement is an absorbing state, $\gamma_{k}^{lags}$ thus gives the accumulated effect of changes in new institutional LTC entitlements per month.

There are a number of issues relevant for the estimation of Equation (1). First, in our context we could have opted to also include individuals who were “never‐treated”, that is, those who were not observed to lose their partner during the observation window.^18^ However, this most likely would then concern individuals who are healthier and/or had different characteristics than those who experienced a partner loss. Namely, having a partner protects against mortality and becoming frail (Kulu et al. 2024). Consequently, using these partners as controls would lead to biased estimates (Goodman‐Bacon 2021). We therefore use only those who will be treated in the future^19^ as a control group.

Second, recent literature argues that the estimation of event‐time models with staggered treatments is problematic when treatment effects change over time (Sun and Abraham 2021; Callaway and Sant’Anna 2021b). Standard OLS estimation of Equation (1) is based on all possible group‐time‐averaged comparisons of individuals' outcome variables at a specific point in time and the actual moment a treatment starts. Each of these combinations gives an “Average Treatment effect on the Treated” (ATT) to obtain estimates of $\gamma_{k}^{lead}$ and $\gamma_{k}^{lags}$. But while comparisons between not‐yet‐treated and treated give consistent estimates, OLS also compares early‐treated and late‐treated. Callaway and Sant’Anna (2021b) show that this could lead to biased estimates of $\gamma_{k}^{lags}$ (and $\gamma_{k}^{lead}$). Callaway and Sant’Anna (2021b) developed an estimation procedure that uses appropriate control groups to separately estimate all relevant ATT effects.^20^ Additionally, they propose different methods to aggregate all ATT effects into single constructs that are easier to use. We use the double robust method to estimate the model, and bootstrapped standard errors as suggested in Callaway and Sant’Anna (2021b). We use the R package especially developed for executing these analyses (Callaway and Sant’Anna 2021a).

Third, a causal interpretation of $\gamma_{k}^{lags}$ in our event‐study model requires the assumption of common pre‐trends for treated and not‐yet‐treated individuals in our sample. This implies that estimates of the pre‐treatment effects $\gamma_{k}^{lead}$ should be equal to zero. We have monthly observations and therefore it will be difficult to completely rule anticipation effects shortly before the death of the partner.

### 3.7 Mortality Bias Correction

There is mortality selection in the data that dynamically changes the composition of the survivors in the sample and hence will also affect the treatment effects. As we showed earlier in Figure 3, individuals who have applied for institutional LTC have a higher mortality rate than those who have not. Consequently, over time, sample attrition due to mortality will mechanically lower the fraction of individuals who apply for institutional LTC. As a result, the currently treated are not comparable to the not‐yet‐treated who have survived up to later times; this will yield estimates that underestimate the impact of partner loss on the need for institutional LTC.

To control for selective mortality attrition, we first derive the resulting bias in the estimate of $\gamma_{k}^{lags}$ $\left(k=1,..,T_{h}\right)$.^21^ We use the observed fraction of institutional LTC applications of the individuals, measured k periods after the moment of partner loss (i.e., $y_{t+k}$). In addition, we use the observed fractions of survivors among those being granted institutional LTC and those not being granted institutional LTC, as measured by k periods after the moment of partner loss. For this we use the average survival rate of individuals; this ensures that survival rates are not influenced by influence of for example seasons on survival of the older individuals in our sample. The survivor rates are defined as $S_{k}^{L}$ and $S_{k}^{N}$, respectively. In the appendix to this paper, we show that the expected value of the estimated lag effect, ${\hat{\gamma }}_{k}^{lags}$, equals the true value plus a bias due to selective mortality:

(2) $$E\left(\;{\hat{\gamma }}_{k}^{lags}\;|\;y_{t+k},S_{k}^{L},S_{k}^{N}\;\right)=\gamma_{k}^{lags}+y_{t+k}\;\left(1-y_{t+k}\right)\;\frac{S_{k}^{L}-S_{k}^{N}}{y_{t+k}S_{k}^{N}+\left(1-y_{t+k}\right)S_{k}^{L}}$$

Equation (2) shows that the bias in $\gamma_{k}^{lags}$ originates from differences in the mortality rates, where individuals are defined as institutional LTC applicants or non‐applicants 1 month before partner loss. Since mortality rates are higher among the initial group of LTC applicants, the share of LTC applicants will mechanically decrease over time. The resulting bias thus depends on the initial share of LTC applicants and the difference in mortality rates. With the observed fractions of $y_{t+k}$, $S_{k}^{L}$ and $S_{k}^{N}$, we can calculate and control for the attrition bias for all relevant values of k. When presenting results of the event‐time model, we show estimates of $\gamma_{k}^{lags}$ that are controlled for this bias.

It is important to stress that mortality bias correction does not only incorporate a priori differences in mortality risks of LTC applicants and non‐LTC applicants, but also differences in the mortality effects of widowhood. For instance, when the impact of widowhood on mortality would be stronger for LTC applicants than for non‐LTC applicants, the effect on LTC applications is absorbed by the bias correction. We therefore interpret $\gamma_{k}^{lags}$ solely as the direct effect of partner loss on institutional LTC entitlement that cannot be taken over by any other informal or formal caregivers.

## 4 Results

### 4.1 Main Analyses: Total Sample and per Somatic and PG Entitlements

**FIGURE 4:**
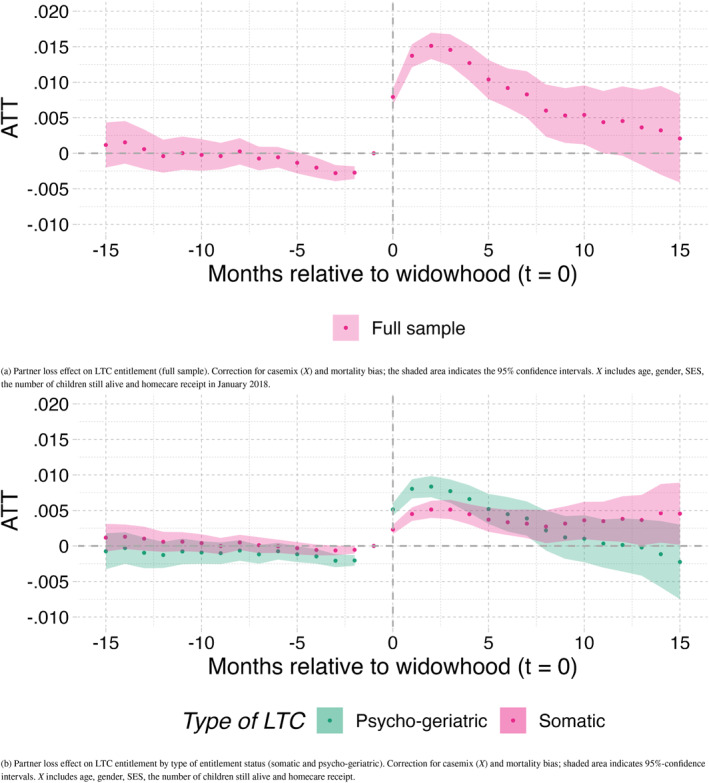
Partner loss effect on all institutional LTC entitlements (panel a) and by type of entitlement (panel b).

Figure 4a plots the ATTs with 95%‐confidence intervals based on the dynamic treatment specification (Equation 1), following the estimation method of Callaway and Sant’Anna (2021b). The estimation results are based on the full sample and corrected for the covariates and for selective mortality (see Equation 2).^22^ The horizontal axis in Figure 4a (and in all results figures below) shows the event time on a monthly resolution, with “0” indicating the month of widowhood. The vertical axis gives the ATT, that is, the effect on the probability of receiving an entitlement for institutional LTC for those who become widowed.

Most notably, we find a peak of the effect of widowhood on institutional LTC needs in the third month after partner loss. At this point, the difference in enrollment between those who have just experienced widowhood and those who have not is 1.5% point; this represents the accumulated effect of higher monthly inflow rates in the first months after partner loss. The effect on LTC needs is short‐lived and almost disappears after around 10 months of widowhood. This suggests that the immediate aftermath after partner loss is the most critical window for increased institutional LTC needs. To further understand the results, it is important to bear in mind that the date used for the outcome in the analyses corresponds to the moment the individual gets an entitlement for institutional LTC, and not the moment that the individual requests it from the CNAC. As stated in the Contextual Background section, the CNAC can take up to 6 weeks to process the application and turn it into an entitlement. This may explain why the peak of the effect is 3 months after partner loss, and not earlier than that.

Figure 4a shows a small decrease in probability of receiving an entitlement in the three to 4 months before partner loss (the maximal probability difference equals 0.25% point). This means that the common pre‐trend assumption for treated and not‐yet‐treated individuals does not seem to hold for the last months prior to partner loss. An explanation for this is that home care workers supporting the frail older individuals, or the individuals themselves, may decide to postpone applications up to the moment of death of the partner. We will further elaborate on this below.

Figure 4b shows the main results separately for somatic and PG entitlements. In our sample, approximately 60% of individuals who receive an institutional LTC entitlement request one for PG care. Following from this, 40% of the entitlements are requests for somatic care. The effect of partner loss appears to be stronger but more short‐lived for individuals with cognitive impairments, such as dementia, as their main health problem. The effect is smaller but more persistent for those with a somatic entitlement. The difference in ATT equals about 0.05% point during the four first months after widowhood between those with PG and somatic health disorders.

**FIGURE 5:**
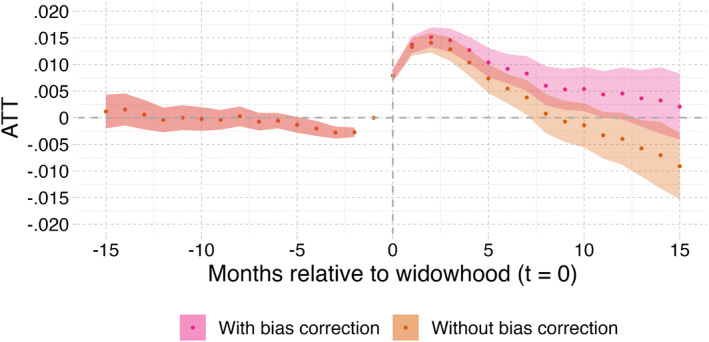
Partner loss effects on full sample: results with and without mortality bias correction. Shaded areas indicate 95%‐confidence intervals. Correction for casemix (X); the shaded area indicates the 95% confidence intervals. X includes age, gender, SES, the number of children still alive and homecare receipt in January 2018.

Finally, Figure 5 displays the results on the full sample with and without the mortality bias correction. It shows that the biasing impact of mortality differences between individuals with and without LTC needs accumulates with the elapsed time after partner loss.^23^ Without correction for selective mortality bias, the effect estimate of widowhood would even fall below zero after 10 months. As stated earlier, it is important to bear in mind that, after correction, we estimate the direct impact of widowhood on the need for institutional LTC care, and not by any differences (indirect) mortality effects.^24^

### 4.2 Mechanisms and Subgroup Analyses

We explained earlier that both the reduced availability of informal care and the worsening of physical and mental health conditions potentially drive the increased needs for institutional LTC (see Figure 1). Unfortunately, the possibilities of investigating these potential mechanisms with mediation analyses are limited; this would require the availability of time‐varying variables, particularly on the health of surviving partners, that capture (part of) the causal effects. This information is limited in the context of our data.^25^

**FIGURE 6:**
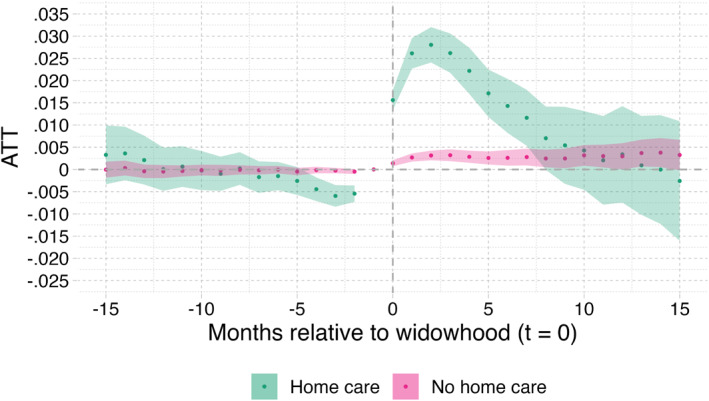
Partner loss effect on LTC entitlement by homecare status (yes/no). Correction for casemix (X) and mortality bias; the shaded area indicates the 95% confidence intervals. X includes age, gender, SES, the number of children still alive and homecare receipt in January 2018.

Having said this, our data do allow to conduct various subgroup analyses that point at important substitution effects of informal care by institutional LTC. To begin with, Figure 6 shows the effects of partner loss on LTC needs by the home care status of the surviving partner. We observe a much stronger effect for the individuals receiving home care during the observation period (up to 2.5% point), than for those who did not. In line with Figure 1, this suggests that widowers with more intensive needs are most likely to receive an entitlement for institutional LTC directly after widowhood. We know that those who receive home care‐like services have significantly more severe health conditions compared to those who do not receive any formal care (Jorm et al. 2010). Figure 6 also displays a drop in applications before widowhood larger than the one in Figure 4a and no drop for those who did not receive home care. This suggests that the deviation from the common trend is mostly explained by those who received home care, that is, those with severe health conditions. Those individuals, or their main formal caregiver, may choose to postpone their CNAC application in the months preceding the partner's death, as they must address serious issues to maintain a delicate balance at home.

**FIGURE 7:**
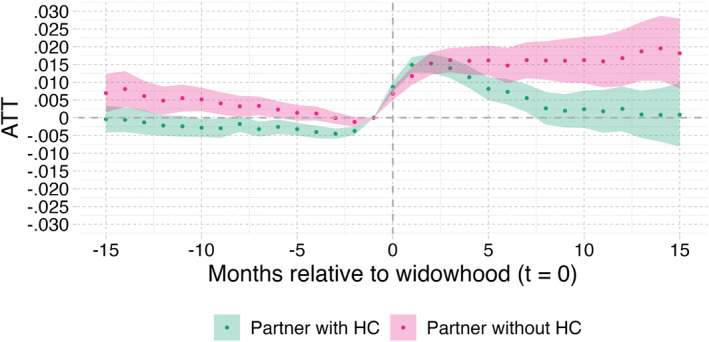
Stratified analyses for individuals whose partners were (not) using home care in Jan. 2018.

Similarly, we performed separate analyses for individuals whose deceased partner required home care before death and those who did not. Figure 7 shows the corresponding results. As explained earlier with the DAG in Figure 1, one would expect the effect of reduced availability of informal care to be strongest for surviving individuals whose partner was sufficiently healthy to provide informal care. The reduced availability of informal care would then generate a direct and potentially transitory effect on institutional LTC needs, which is confirmed by our results for the full sample. We observed that the effect on the surviving spouse was much more persistent when the deceased partner was not receiving any home care (comprising 32% of our sample). Since partners without home care were most likely in relatively good health, and therefore more able to provide more help than those with home care, this suggests that the reduced availability of informal care is indeed an important driver of our results. We further elaborate on this in Section 5.

**FIGURE 8:**
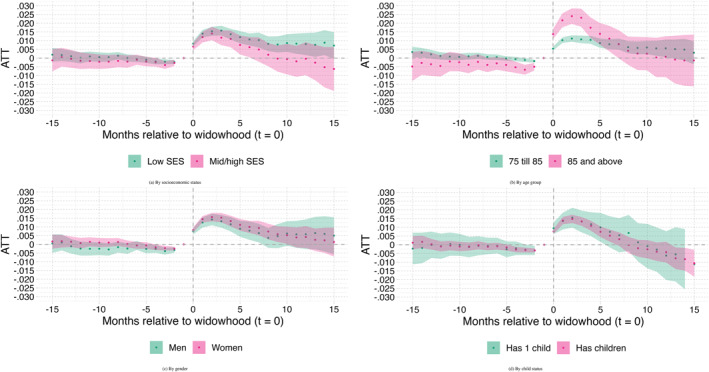
Subgroup results of partner loss effect on LTC entitlement. Correction for casemix (X) and mortality bias; the shaded area indicates the 95% confidence intervals. X includes age, gender, SES, the number of children still alive and homecare receipt in January 2018.

**FIGURE 9:**
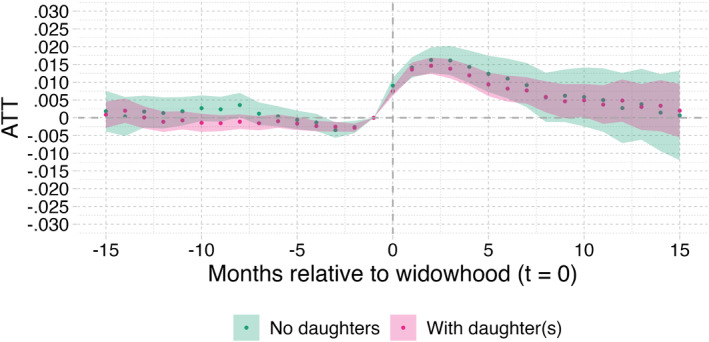
Stratified analyses for individuals with and without daughters at January 2018. Correction for casemix (X) and mortality bias; the shaded area indicates the 95% confidence intervals. X includes age, gender, SES, the number of children still alive and homecare receipt in January 2018.

Finally, the panels in Figure 8 show the estimation results by SES, age, gender and child status.^26^ We observe in panel (a) that the effect on needs for institutional LTC is not significantly different for the individuals with a lower SES than for those with a higher SES, since the confidence intervals of the two groups completely overlap. However, the effect lasts slightly longer for individuals with a lower SES, namely 7 months, compared to those with higher SES, for whom the effect lasts 4 months. Panel (b) shows that individuals older than 85 are much more impacted by widowhood than individuals aged 75–85, but that the effect is longer lasting for the younger individuals (those aged 75–85). The difference between the two groups is equal to about 1.5% point during the first 4 months after widowhood, and is statistically significant. At odds with expectations, the results shown in panel (c) do not point at any statistical differences in ATTs between men and women. This for instance disagrees with Katsiferis et al. (2023) who found the impact of widowhood on mortality to be significantly different for men compared to women. We also expected stronger effects among men, given that men from these generations typically are less proficient in household tasks and women have traditionally taken care of daily life responsibilities. Partner loss could therefore lead to men facing more difficulties with taking care of themselves and to men developing more quickly needs for institutional LTC. Finally, panel (d) shows the results separately for the group with more than one child alive on January 1, 2018 and for the group with only one child alive at January 1, 2018. Again, we do not see any meaningful differences between the two groups.^27^ This result was also unexpected, as individuals with more children are likely to be able to maintain “aging‐in‐place” longer than those with only one child. To further explore the role of children after spousal bereavement, we performed additional analyses on those with at least one daughter and those without, as daughters are more likely to provide informal care than sons. As shown in Figure 9, these analyses did not reveal significant differences in the likelihood of accessing institutional LTC after spousal bereavement between households with at least one daughter and those without.

### 4.3 Sensitivity Analyses

**FIGURE A1:**
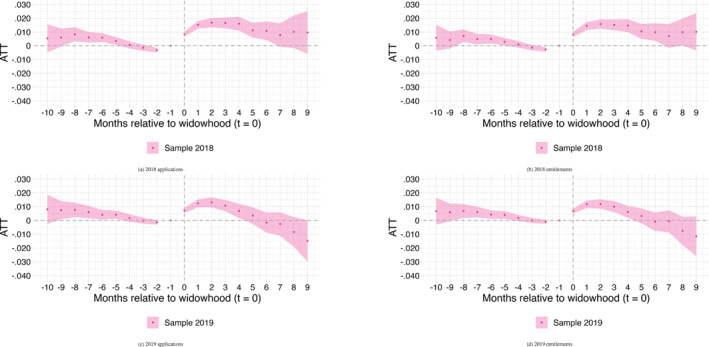
Partner loss effects for years 2018 and 2019 analyzed for two outcomes separately. All four are corrected for casemix (X) and mortality bias; the shaded areas indicate 95%‐confidence intervals. X includes age, gender, SES, homecare receival status and the number of children alive. The vertical axis gives the ATT, that is, the effect on the probability of receiving an entitlement for institutional LTC for those who become widowed.

**FIGURE A2:**
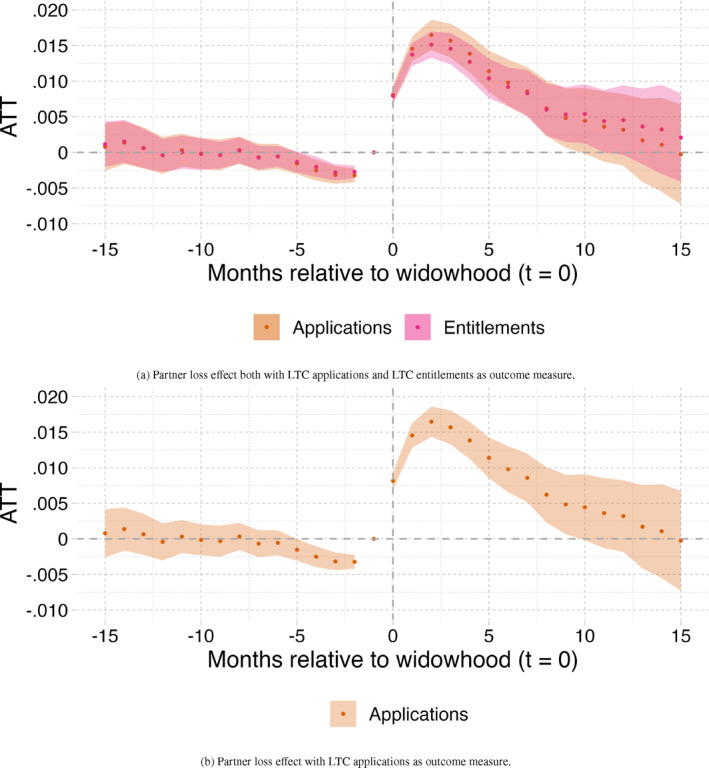
Partner loss effects analyzed separately for two outcomes: LTC applications and entitlements. Both are corrected for casemix (X) and mortality bias; the shaded areas indicate 95%‐confidence intervals. X includes age, gender, SES, homecare receival status and the number of children alive. The vertical axis gives the ATT, that is, the effect on the probability of receiving an entitlement for institutional LTC for those who become widowed.

To test for the robustness of our findings, we estimated alternative model variants; the outcomes are all presented in Appendix C. To check for yearly cohort differences, we estimated our main models separately for individuals who became widowed in the year 2018 and in 2019, respectively, see Figure A1. This indicates that the individuals who lose their partner in the first half of our observation period do not behave differently than the individuals who lose their partner in the second half. Second, we estimated the main result for the full sample using applications for LTCa entitlement as outcome instead of the LTCa entitlements (objectively reviewed and approved applications by CNAC). These results are shown in Figure A2. We find that using applications for LTCa as an outcome generally yields similar results. This indicates that the vast majority of individuals who lose a partner, and applies for institutional LTC, got an entitlement. Overall, the results of these sensitivity analyses confirm our main results.

## 5 Discussion and Conclusion

We study the impact of partner loss on the onset of needs for institutional LTC. Based on the existing literature, this impact can be twofold. First, since spouses are at the forefront when it comes to providing informal care, widowed individuals have to rely on institutional LTC when the demand for care cannot be taken over by other (formal or informal) sources of care. Second, the loss of a partner may cause adverse health consequences, potentially resulting in needs for institutional LTC (Pena‐Longobardo et al. 2021; Liu et al. 2019; Carey et al. 2014; van Boekel et al. 2021; Zhao et al. 2021; van den Berg et al. 2011).

Our main results show that partner loss significantly increases the likelihood of receiving an entitlement for institutional LTC. The average effect of partner loss on the need for institutional LTC peaks at a 1.5% point rise 3 months after widowhood and disappears after 10 months. The effect is slightly stronger for individuals with PG care, and much stronger for those who were receiving home care before partner loss and for the oldest individuals. All of this suggests that those who are likely to receive an entitlement for institutional LTC shortly after the loss of their partner are the ones who were already frail before partner loss. When both partners are vulnerable, they probably manage aging‐in‐place by providing each other support, in many cases together with some types of formal home care. Eventually, once one of them passes away, it is no longer possible to maintain aging‐in‐place, and the remaining partner has to apply for an entitlement for institutional LTC. In addition, the absence of significant difference between men and women suggests that not gender, but health status of the widowed individual drives most of increase in institutional LTC needs.

In addition, we find that the effects persist only for survivors whose partners did not receive home care before their death, suggesting that the reduced availability of informal care is an important driver of the increase in institutional LTC needs. This again points at substitution of informal by formal care as a major driver of our results, rather than bereavement effects on physical and mental health that evolve and accumulate over time. With the latter mechanism, the effects on entitlements should persist and accumulate over time.

Our evidence suggests an increase in the overall volume of use of institutional LTC as well. In an attempt to quantify this volume, we use Figure 4a, and compute the number of additional months with entitlement per year due to widowhood. This is equal to about 2620 months since it can be approximated as the area under the curve times the total number of widowers per year still living at home (10.7%* 24,500). However, it should be noted that this does not automatically translate into 2620 additional months of use of institutional LTC.^28^ There are two main reasons for this. First, individuals typically experience a waiting period between receiving an entitlement and the actual use institutional LTC. This duration from entitlement until start of institutional LTC use is not observed in the data available for this study. Second, as mentioned above, we cannot fully rule out the possibility that losing a partner positively impacts the mortality rate of the widowed individual, partly because of adverse health consequences, for example (van den Berg et al. 2011). As a result, we cannot compute the number of additional months of actual use of institutional LTC due to widowhood. Although considering entitlements provides valuable information on the development of needs for institutional LTC, we recommend also investigating the actual use of care in future research, as it may provide additional information.

At the time of the study, we had no access to information on the cause of the partner's death or on most characteristics of the children. The nature of a partner's passing—whether sudden or following a prolonged illness—may influence the widowed individual's need for institutional LTC. With data on the duration and nature of the illnesses leading to death, we could have more specifically analyzed the anticipation effects for widowed individuals who do or do not experience partner's prolonged illness before the loss. Similarly, we could not analyze the role of the children's age, employment status or how far they live from their (widowed) parents in the process of demanding institutional LTC after widowhood.

Finally, this study does not provide information on those living alone, as they were excluded from our study sample. An increasingly larger group of individuals aged 75 and above live alone (van Duin et al. 2018). These individuals can be broadly categorized into two distinct groups. One group encompasses those consciously choosing to live alone. On average, this group experiences almost no loneliness and has a large social network by fostering close relationships with family, friends, or neighborhood. As a result, they may rely less frequently on institutional LTC than others. However, the second group is involuntarily living alone. This group will on average experience more loneliness and has a smaller social network. We expect this group to rely on institutional LTC from quite early on when compared with the first group. It is unclear how this plays a role in the further development of the burden on the (institutional) LTC system.

To conclude, our study shows substantial positive effects of widowhood on the needs for institutional LTC, which will partly result in an additional use of expensive care. A relevant policy question relates to the desirability and feasibility of postponing needs for institutional LTC after widowhood. Maintaining these vulnerable individuals at home will necessitate further, potentially unrealistic, support from for example, children or other family members as well as already overburdened formal caregivers. Increasing caregiver resilience may provide some relief, but for frail older individuals (home care receivers), this is rarely sufficient. Children may not always live nearby and often have demanding careers. We know from recent research by Korfhage and Fischer‐Weckemann (2024), that informal caregiving by children is associated with significantly worse labor‐market opportunities. Additionally, children have their own family commitments. While alternatives like community care can offer assistance in certain aspects, they rarely offer round‐the‐clock supervision, that partners often provide. Investigating whether it is desirable and cost‐effective to maintain frail older individuals at home after widowhood is therefore a topic for future research.

## Conflicts of Interest

The authors declare no conflicts of interest.

## Data Availability

The data that support the findings of this study are available from Statistics Netherlands under certain conditions. Restrictions apply to the availability of these data, which were used under license for this study. Data are available from https://www.cbs.nl/en‐gb/our‐services/customised‐services‐microdata/microdata‐conducting‐your‐own‐research with the permission of Statistics Netherlands under certain conditions.

## Endnotes

^1^
Formal home care is defined as health or supportive care provided by a professional caregiver in the home setting.

^2^
In OECD countries, half of the LTC expenditures is spent on care in nursing homes, in the Netherlands this amounts to over 80% of LTC expenditures (OECD 2023c).

^3^
In 2018, 44% of the individuals who entered institutional LTC with a light PG profile and 52% of the individuals who entered with a more severe PG profile lived longer than two years in a nursing home. In comparison, in 2018, 37% of the individuals who entered institutional LTC with a light somatic profile and 31% of the individuals who entered with a more severe somatic profile lived longer than two years in a nursing home (ten Koppel et al. 2023).

^4^
Home care is defined as personalized care assistance provided by a district nurse (see the Contextual Background for more details). As such, it is an indicator of the severity of the individual's health condition.

^5^
Note that frailty and care needs vary significantly among older people. Therefore, the duration in which an individual progresses through the stages of the care pathway and the extent to which the individual utilizes care in these stages can vary widely.

^6^
In the Netherlands, there is virtually no private institutional LTC. Even when institutional LTC providers are privately owned, clients still require approval from the gatekeeper to access such care. Without this approval, individuals are not eligible for publicly‐insured institutional care and would face prohibitively high costs that few can afford.

^7^
In theory, individuals who have been granted access to round‐the‐clock care might have a choice between remaining at home or in home‐like settings, or moving to a nursing home. However, this choice is influenced by several factors such as the available supply of home care facilities, advice from healthcare professionals and family members. In addition, if the expected costs of living at home exceed those of living in a nursing home, individuals are required to move to a nursing home.

^8^
For PG care, dementia is the most common diagnosis.

^9^
Those aged 75 and over are most at risk of receiving institutional LTC. We excluded those over 100, as the information for these individuals was occasionally unreliable.

^10^
It can also be for example a sibling or a friend.

^11^
We explain this selection criterion in more detail when we present the empirical model.

^12^
We explain the reason for this time‐frame later on.

^13^
Privacy regulation requires that average income information can not be provided if there are too few people living in the 4‐digit postal code.

^14^
A simple comparison of couples who lose a partner with those who do not lose a partner would induce a form of selectivity because these two types of households may be structurally different.

^15^
In the Netherlands, all individuals who want to have access to round‐the‐clock care need an entitlement from the gatekeeper (CNAC). Because of that, our main dependent variable which takes the value of one for individuals who have received a CNAC entitlement measures access to round‐the‐clock care provided either in a nursing home, at home or in homelike settings.

^16^
We use the values of the covariates at the start of the observation period.

^17^
Age, gender and the home care variable are included in the analyses to control for initial differences in health of the surviving partner.

^18^
Recall that we do not include individuals who had already lost their spouse at January 1, 2018 (i.e., the “already treated”).

^19^
That is, the “not‐yet‐treated” who had not yet lost their partner when the treated individual was observed to lose his/her partner.

^20^
Note that in our case, we have 24 treatment periods (i.e., 24 groups of treated individuals) and we end up with 276 different ATT's for all possible comparisons of time periods.

^21^
Appendix B shows the derivation and the comparison of the results with and without bias correction.

^22^
Note again that all graphs shown in this section are based on regressions that employ the use of district nursing care, age, and gender as controls for health status of the surviving partner. When extending this set with additional information on the dementia status of individuals and their annual total medical health insurance costs in 2017, our results are virtually equivalent in all cases.

^23^
Recall that mortality bias correction also incorporates differences in causal effects of widowhood on the mortality between LTC‐applicants and non‐LTC applicants.

^24^
Knowing that mortality rates are around six times higher for individuals with institutional LTC needs than those without, it is plausible that a major part of the bias correction can be interpreted as ex ante differences in mortality, and not as differences in mortality effects of widowhood that come on top of this.

^25^
Moreover, the implementation of Callaway and Sant’Anna (2021b) does not allow accommodating and controlling for time‐varying variables.

^26^
We also performed stratified analyses per type of location (i.e., rural vs. urban), but we could not find any significant differences.

^27^
The group of individuals without children at January 2018 was very small. We therefore could not run meaningful separate analyses for those with and without living children.

^28^
On average,1 month of institutional LTC costs 7300 euros (Krabbe‐Alkemade et al. 2020).

## Appendix A Share and Type of Informal Care Provided by Family Members

We have used data from the Longitudinal Aging Study Amsterdam (LASA) to shed light on informal care (extensive margin) provided by family members to older Dutch individuals. The LASA study is a multidisciplinary ongoing cohort study initiated in 1992 that includes information related to the physical, cognitive, emotional, and social well‐being of older adults (Deeg et al. 1993). Specifically, we used data from the most recent wave (2021–22) to construct Table A1 below. The table shows that partners provide most informal care (up to 77% of personal and nursing care) while children (biological and in‐law) mainly help with domestic tasks, guidance outside the home and administrative support.

**TABLE A1:** Proportion of care provided informally or formally by type of care.

	Personal care	Domestic care	Nursing care	Guidance ^a^	Administration
Informal care:					
Partner (%)	24.1	11.5	21.4	30.1	9.6
Children (biological and in‐law) (%)	7.0	23.2	4.7	61.3	57.4
Other sources of informal^b^ (%)	0	17.1	0	46.8	13.0
Formal care (%)	72.2	42.1	71.4	19.3	5.2

^a^ Help outside the house.

^b^ Care from other family members, neighbors, friends or volunteers.

## Appendix B Mortality Bias Derivation

In this appendix we derive the attrition bias due to selective mortality on the estimated effect of partner loss on the probability of institutional LTC applications, $\gamma_{k}^{lags}$. We show that differences in mortality between LTC applicants and non‐LTC‐applicants lead to a mechanical and accumulating effect that is expected to lower the fraction of LTC applications over time, as compared to the initial fraction at the moment of partner loss t.

We depart from Equation (1), and abstract from the effect of calendar‐time effects ϕ. We denote $y_{t}$ and $y_{t+k}$ as the observed share of individuals with an LTC application at time t and at time t+k.^29^ In this context, the “event‐time” estimate of $\gamma_{k}^{lags}$ (with $k=1,..,T_{h}$) without controlling for selective mortality bias equals the difference between shares of LTC applicants between t+k and t:

(A1)
$${\hat{\gamma }}_{k}^{lags}=y_{t+k}-y_{t}$$

We next introduce the observed survivor rates $S_{k}^{L}$ and $S_{k}^{N}$ until time t+k for the cohorts of individuals at the moment of partner loss t with and without LTC applications, respectively. Any differences between $S_{k}^{L}$ and $S_{k}^{N}$ affect $y_{t+k}$ and therefore lead to biased estimation of $\gamma_{k}^{lags}$. We define ${\tilde{y}}_{t+k}$ as the share of LTC applicants k periods after partner loss t that would have resulted without mortality attrition. For this, we rescale the number of LTC applicants and non‐LTC applicants by the inverse of their corresponding survival rates:

(A2)
$$\begin{array}{ll} {\tilde{y}}_{t+k} & =\frac{y_{t+k}\;/S_{k}^{L}}{y_{t+k}\;/S_{k}^{L}+\left(1-y_{t+k}\right)\;/S_{k}^{N}}\\ & =\frac{y_{t+k}\;S_{k}^{N}}{y_{t+k}\;S_{k}^{N}+\left(1-y_{t+k}\right)\;S_{k}^{L}} \end{array}$$

For consistent estimation of $\gamma_{k}^{lags}$, we compare the counterfactual outcome $y_{t}$ to ${\tilde{y}}_{t+k}$, since this outcome is not susceptible to selective mortality bias (as $y_{t+k}$). The biasing impact of selective attrition, defined as $\Delta_{k}$, is thus equal to the difference between $y_{t+k}$ and ${\tilde{y}}_{t+k}$ and can be written as a function of $y_{t+k}$, $S_{k}^{L}$, and $S_{k}^{N}$:

(A3)
$$\begin{array}{ll} \Delta_{k}=y_{t+k}-{\tilde{y}}_{t+k} & =\frac{y_{t+k}\left(y_{t+k}S_{k}^{N}+\left(1-y_{t+k}\right)S_{k}^{L}\right)-y_{t+k}S_{k}^{N}}{y_{t+k}S_{k}^{N}+\left(1-y_{t+k}\right)S_{k}^{L}}\\ & =y_{t+k}\cdot \left(1-y_{t+k}\right)\cdot \frac{S_{k}^{L}-S_{k}^{N}}{y_{t+k}S_{k}^{N}+\left(1-y_{t+k}\right)S_{k}^{L}} \end{array}$$

We next rewrite Equation A1 and insert $\Delta_{k}$:

(A4)
$${\hat{\gamma }}_{k}^{lags}=\left({\tilde{y}}_{t+k}-y_{t}\right)+\Delta_{k}$$

Conditional on $y_{t+k}$, $S_{k}^{L}$ and $S_{k}^{N}$, the expected value of the estimand ${\hat{\gamma }}_{k}^{lags}$ thus equals

(A5)
$$E\left({\hat{\gamma }}_{k}^{lags}\;|\;y_{t+k},S_{k}^{L},S_{k}^{N}\;\right)=\gamma_{k}^{lags}+\Delta_{k}$$

Equation A5 shows that we can use the observed values of $y_{t+k}$, $S_{k}^{L}$ and $S_{k}^{N}$ in our sample to calculate Δ(k), that is the biasing impact of selective attrition on $\gamma_{k}^{lags}$. By subtracting the bias from the initial estimate of $\gamma_{t+k}^{lags}$, we re‐scale the value of $y_{t+k}$ to control for the effect of selective attrition. Since mortality rates of LTC applicants will generally exceed those of non‐applicants $\left(S_{k}^{L}<S_{k}^{N}\right)$, selective attrition most likely has a downward impact on the fraction of LTC applicants. Consequently, the estimate of $\gamma_{k}^{lags}$ will be underestimated without controlling for the bias.
